# L-3-[^18^F]-Fluoro-α-Methyl Tyrosine as a PET Tracer for Tumor Diagnosis: A Systematic Review from Mechanisms to Clinical Applications

**DOI:** 10.3390/ijms26125848

**Published:** 2025-06-18

**Authors:** Mei Bao, Xiang Gu, Kai Tong, Fei Chu, Pinmao Ye, Kazuko Kaneda-Nakashima, Wenbin Hou, Yiliang Li, Ling Wei

**Affiliations:** 1 School of Chinese Materia Medica, Guangdong Pharmaceutical University, Guangzhou 510006, China; meibao678@163.com (M.B.); gx18834153125@163.com (X.G.); 13118762620@163.com (K.T.); 15555668056@163.com (F.C.); pinmao13@163.com (P.Y.); 2 Division of Radiation Science, Institute for Radiation Sciences, Osaka University, 2-4 Yamadaoka, Suita 565-0871, Osaka, Japan; kanedak17@chem.sci.osaka-u.ac.jp; 3 Core for Medicine and Science Collaborative Research and Education, Forefront Research Center, Graduate School of Science, Osaka University, 1-1 Machikaneyama, Toyonaka 560-0043, Osaka, Japan; 4 Tianjin Key Laboratory of Radiation Medicine and Molecular Nuclear Medicine, Institute of Radiation Medicine, Chinese Academy of Medical Sciences and Peking Union Medical College, Tianjin 300192, China; houwenbin@irm-cams.ac.cn (W.H.); liyiliang@irm-cams.ac.cn (Y.L.)

**Keywords:** [^18^F]FAMT, amino acid PET, LAT1, molecular imaging, tumor diagnosis, treatment response assessment

## Abstract

L-3-[^18^F]-fluoro-α-methyl tyrosine ([^18^F]FAMT) is an amino acid positron emission tomography (PET) tracer with high specificity for malignant tumors through its selective transport via L-type amino acid transporter (LAT) 1. Although extensively studied for its diagnostic performance, a comprehensive review of its molecular and clinical characteristics remains lacking. A systematic literature review (1997–2025) was conducted using PubMed and Web of Science, with keywords including “L-3-[^18^F]-fluoro-α-methyl tyrosine”, “[^18^F]FAMT”, “amino acid PET”, and “tumor imaging”. The review covered aspects of synthesis, structural properties, pharmacokinetics, and clinical applications. Notably, while research on [^18^F]FAMT has declined significantly in recent years, [^18^F]FAMT PET demonstrates superior specificity to [^18^F]FDG PET in distinguishing malignancies from inflammatory lesions and offers distinct advantages in lung, esophageal, and oral cancers, though with slightly lower sensitivity. Its key features include tumor-specific uptake patterns, rapid blood clearance, and a significant correlation between its uptake levels and both LAT1 expression and tumor proliferation. In conclusion, [^18^F]FAMT is a promising PET tracer with notable advantages in tumor imaging, particularly due to its LAT1 selectivity and favorable pharmacokinetics. Despite challenges in production, these characteristics underscore its clinical value in cancers requiring precise imaging. Future research should focus on optimizing synthesis, expanding clinical validation, and exploring theranostic applications.

## 1. Introduction

Accurate tumor diagnosis is crucial for effective cancer management. Over the past two decades, radioactive tracers have emerged as powerful tools for visualizing abnormal metabolic changes in tumors, revolutionizing the field of oncological imaging [1,2]. Among these, positron emission tomography (PET) tracers have shown remarkable success in detecting and characterizing various malignancies [3]. The glucose analog 2-[^18^F]Fluoro-2-deoxy-D-glucose ([^18^F]FDG) has been the most commonly used PET tracer [1], accumulating in tumor cells via glucose transporters on the plasma membrane [4,5]. However, [^18^F]FDG PET has well-recognized limitations, including high uptake in inflammatory lesions and granulation tissues, as well as high physiologic background in some normal tissues such as the brain, leading to false-positive diagnostic findings [6,7].

To overcome these disadvantages, radiolabeled amino acids with great tumor selectivity have been developed [8]. L-methyl-^11^C-methionine (^11^C-MET) has been a well-established amino acid PET tracer, but its short half-life of ^11^C restricts its use to PET centers with on-site cyclotrons [9]. Subsequently, ^18^F-labeled amino acid radiotracers, such as O-(2-[^18^F]-fluoroethyl)-L-tyrosine([^18^F]FET), [^18^F]-fluoro-L-dihydroxy phenylalanine([^18^F]FDOPA), were introduced, though these still suffer from some physiological backgrounds and false positives in PET [10,11].

In the realm of single-photon emission computed tomography (SPECT), the amino acid tracer 3-[^123^I]-L-α-methyl-tyrosine ([^123^I]IMT) demonstrated usefulness in tumor imaging with fewer false positives and low brain-background [12]. Building on this success, L-3-[^18^F]-fluoro-α-methyl tyrosine ([^18^F]FAMT) was developed as a PET tracer by replacing ^123^I with ^18^F [13]. [^18^F]FAMT has emerged as a promising PET tracer, exhibiting higher cancer specificity in peripheral tissues compared to other amino acid PET tracers and [^18^F]FDG [6,14]. This specificity stems from its selective transport via L-type amino acid transporter 1(LAT1) [14,15], which is predominantly expressed in cancer cells [16]. Notably, [^18^F]FAMT accumulates in tumors without incorporation into protein synthesis [13,14,17], contributing to its distinctive imaging characteristics. Despite these promising features, the full potential and limitations of [^18^F]FAMT in clinical practice are still being explored. Questions remain about its diagnostic applicability across different types of cancer, its role in treatment monitoring, and how it compares to newer PET tracers in development.

The aim of this review is to comprehensively examine [^18^F]FAMT from its molecular foundations to clinical applications in tumor diagnosis. This review covers its structural design, transport mechanisms, diagnostic performance across various cancer types, and comparative advantages over other PET tracers, while also addressing current challenges and future perspectives in oncological imaging. By integrating and analyzing recent studies, we aim to provide clinicians and researchers with a thorough understanding of [^18^F]FAMT’s role in modern cancer diagnostics and its potential future applications.

## 2. Methods

This systematic review adhered to the Preferred Reporting Items for Systematic Reviews and Meta-Analyses (PRISMA) guidelines; however, it was not registered [18].

This review focused on [^18^F]FAMT studies in oncology published between 1997 and 2025. A comprehensive literature search was conducted in PubMed and Web of Science databases using the terms “[^18^F]FAMT”, “L-3-[^18^F]-fluoro-α-methyl tyrosine”, “3-fluoro-L-α-methyl-tyrosine”, and “FAMT”. Additional relevant records were identified through reference list screening and expert recommendations.

**Figure 1:**
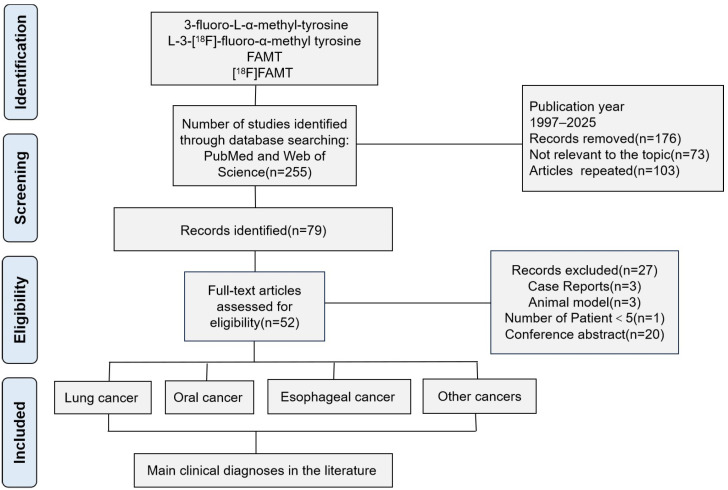
[^18^F]FAMT flowchart of the article selection process.

The initial search yielded 255 records. After removing 103 duplicates, 152 unique records remained for screening. Following the title and abstract review, 73 records were excluded due to irrelevance. A full-text assessment was conducted on the remaining 79 articles, of which 27 were subsequently excluded. The complete article selection process is illustrated in Figure 1.

**Figure 2:**
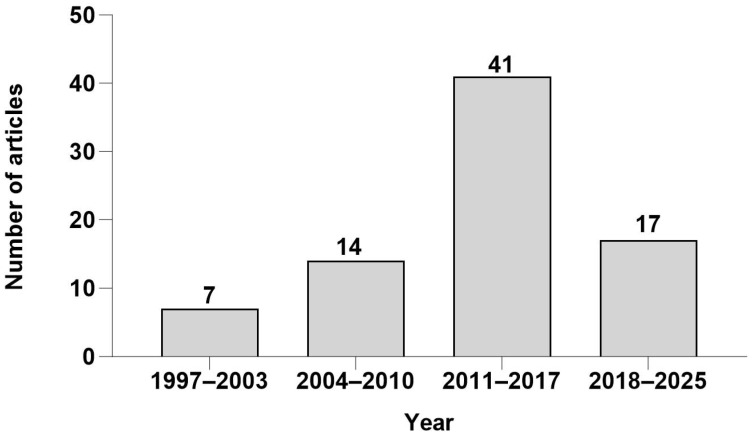
Number of articles for [^18^F]FAMT from 1997 to 2025.

Research on [^18^F]FAMT spans nearly 30 years since its initial development in 1997 [13]. The number of publications showed steady growth and reached its peak during 2011–2017 with 41 articles. Although the publication rate has moderated in recent years, [^18^F]FAMT continues to attract research interest as a promising PET tracer, particularly for its high cancer specificity in tumor imaging (Figure 2).

## 3. Results and Discussion

### 3.1. Radiosynthesis of [^18^F]FAMT

The radiosynthesis of [^18^F]FAMT primarily involves two methodologies: direct and indirect labeling. The direct method entails electrophilic [^18^F]-fluorination, typically without altering the molecular carbon skeleton, while the indirect method involves stepwise synthesis from small precursors, generally incorporating [^18^F]fluoride via nucleophilic substitution reactions [19].

Both methods have their advantages and limitations. The direct method typically requires protection of reactive functional groups and may necessitate additional steps, such as reduction or oxidation, following fluorination [20]. In contrast, the indirect method, though more complex, allows for the labeling of biologically relevant molecules that are incompatible with the conditions required for direct ^18^F-fluorination conditions [19].

Tomiyoshi et al. established a production and purification process for [^18^F]FAMT based on direct electrophilic fluorination, achieving approximately 20% radiochemical yield with a radiochemical purity of ~99% [13]. The process began with the fluorination of [^18^F]F2 to ^18^F-acetylhypofluorite (CH_3_COO^18^F), followed by its introduction into a solution containing L-α-methyl tyrosine in trifluoroacetic acid under ice-water cooling. After fluorination and solvent evaporation, the reactants were dissolved in water. Although this method enhances radiation safety and reproducibility, the overall radiochemical yield remains suboptimal. In clinical practice, direct electrophilic fluorination of [^18^F]FAMT yields a small number of positional isomers, primarily 2-[^18^F]FAMT, alongside the predominant 3-[^18^F]FAMT [9]. Despite the trace presence of 2-[^18^F]FAMT, [^18^F]FAMT is routinely applied without isomeric separation, due to the negligible proportion of 2-[^18^F]FAMT and the lack of reported impact on safety or diagnostic performance.

### 3.2. Structural Characteristics of [^18^F]FAMT

[^18^F]FAMT belongs to a family of ^18^F-labelled amino acid tracers developed to overcome the limitations of ^11^C-labelled tracers such as ^11^C-Methionine [21]. The key limitation of ^11^C-Methionine lies in its shorter half-life and inability to be stored for extended periods, which has driven the development of various ^18^F-labelled amino acid tracers, including O-^18^F-fluoromethyl-L-tyrosine [22].

**Figure 3:**
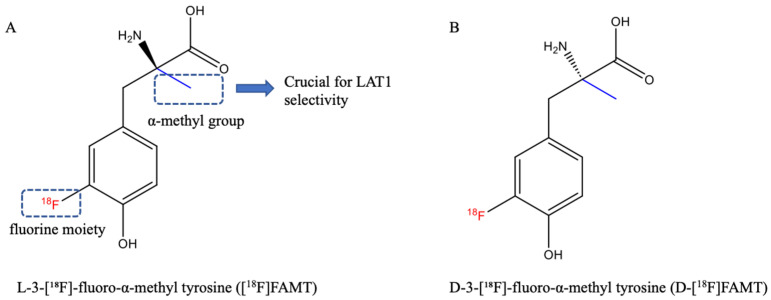
Structural characteristics of [^18^F]FAMT (**A**) and D-[^18^F]FAMT (**B**).

The molecular structure of [^18^F]FAMT features two critical components: an α-methyl group and a fluorine moiety at the 3-position of the aromatic ring (Figure 3A). Structure–activity relationship studies have revealed crucial insights into the importance of these structural elements [23]. While structural analogs 3-fluoro-L-tyrosine (3-FT) and 2-fluoro-L-tyrosine (2-FT), containing fluorine moieties but lacking α-methyl groups, are transported by both LAT1 and LAT2 (similar to L-tyrosine), L-α-methyltyrosine (AMT), lacking fluorine but retaining the α-methyl group, maintains LAT1 selectivity. This evidence confirms that the α-methyl group, rather than the fluorine moiety or its position, is the crucial structural determinant for LAT1 selectivity.

[^18^F]FAMT can also be synthesized in D-isomeric form (Figure 3B), which exhibits distinct biological properties from the L-isomer [24,25]. The D-isomer demonstrates high stability both in vitro and in vivo. While maintaining tumor uptake capability, D-[^18^F]FAMT shows unique pharmacokinetic properties, including rapid blood clearance and predominantly urinary excretion, leading to minimal accumulation in non-target organs. Despite these advantages in background reduction, the tumor accumulation level of D-[^18^F]FAMT is lower than that of L-[^18^F]FAMT, and research on D-[^18^F]FAMT remains limited.

### 3.3. Molecular Mechanism: [^18^F]FAMT and LAT1 Interaction

**Figure 4:**
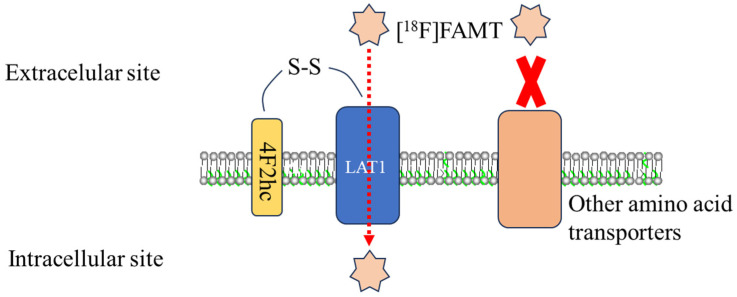
[^18^F]FAMT transport via L-type amino acid transporter 1. [^18^F]FAMT (represented by star symbols) is selectively transported across the cell membrane by LAT1 (blue). LAT1 forms a functional heterodimeric complex with 4F2hc (yellow) via a disulfide bond (S–S), enabling the specific uptake of [^18^F]FAMT from the extracellular to the intracellular compartment (red dotted arrow). In contrast, other amino acid transporters (orange) do not significantly contribute to [^18^F]FAMT uptake, as indicated by the red X, highlighting the high substrate selectivity of this radiotracer for LAT1-mediated transport.

LAT1, a major nutrient transport system, mediates the sodium-independent uptake of various large neutral amino acids, such as leucine, phenylalanine, tyrosine, etc., which are essential for cellular growth and proliferation [26]. The functional expression of LAT1 in the plasma membrane requires its covalent binding to the heavy chain of 4F2 cell surface antigens (4F2hc), forming a heterodimeric complex via a disulfide bond [27,28]. While 4F2hc is ubiquitously expressed in various tissues, LAT1 expression shows a highly restricted pattern, primarily limited to tissues with specific amino acid transport requirements such as the brain, placenta, and testis under normal physiological conditions [29,30]. LAT1 is highly expressed in both primary human tumors and cultured cancer cell lines, where it provides essential amino acids to support rapid tumor cell proliferation [31,32,33]. This upregulation of LAT1 in malignant tissues, combined with its limited expression in normal tissues, provides the molecular basis for [^18^F]FAMT tumor-specific accumulation (Figure 4) [15].

The transport selectivity of [^18^F]FAMT is notable among amino acid PET tracers. Unlike other tracers that may be transported by multiple amino acid transport systems, [^18^F]FAMT uptake is specifically mediated by LAT1 [34,35], with minimal interaction with LAT2 [14,36]. This selective transport mechanism, coupled with the fact that [^18^F]FAMT is not metabolized intracellularly due to its α-methyl group, results in lower background accumulation in organs such as the pancreas and liver, enhancing its value in tumor imaging [37].

Clinical studies have demonstrated strong correlations between [^18^F]FAMT uptake, LAT1 expression, and tumor proliferation markers. The SUV_max_ of [^18^F]FAMT demonstrated consistent correlation with both LAT1 expression and Ki-67 indices, providing a molecular basis for its utility in assessing tumor aggressiveness [38,39].

### 3.4. Pharmacokinetics of [^18^F]FAMT

[^18^F]FAMT exhibits distinctive pharmacokinetic characteristics that contribute to its diagnostic performance as a tumor imaging agent.

**Figure 5:**
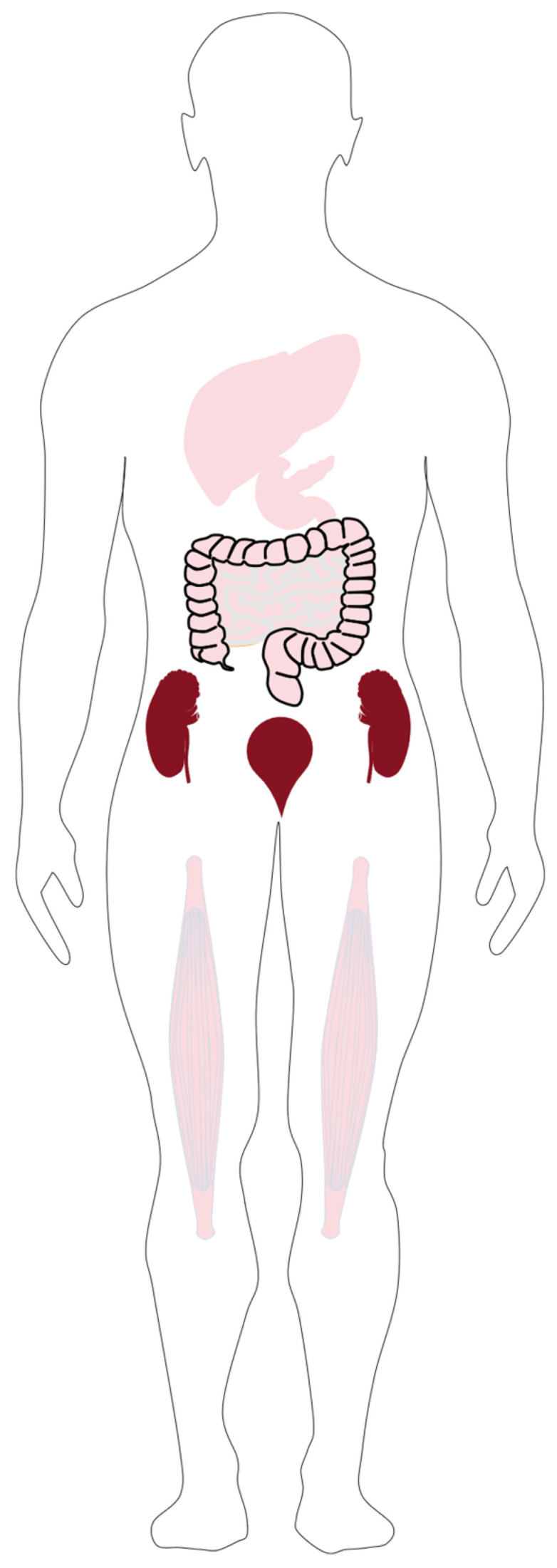
Anatomical biodistribution profile of [^18^F]-FAMT.

In humans, [^18^F]FAMT demonstrates remarkable metabolic stability, with unmetabolized tracer accounting for over 99.5% of plasma radioactivity up to 60 min post-injection [40]. Human studies show rapid plasma clearance (t_₁/₂_ ≈ 3 min), efficient urinary excretion (approximately 50% of injected dose within 60 min), and low physiological uptake in normal brain tissue (SUV~1.6) [40]. The in vivo biodistribution is characterized by significant uptake in the urinary bladder, high accumulation with slow clearance in the kidneys, and elevated accumulation in the pancreas, while lower accumulation levels are observed in the liver, intestines, stomach, and muscle tissue, as shown in Figure 5 [24,40,41]. Brain uptake kinetics show maximal accumulation between 15 and 25 min post-injection, followed by washout [40]. 

Preclinical studies in rodents showed [^18^F]FAMT achieves favorable tumor-to-blood (>1.6) and tumor-to-muscle (>2.8) ratios maintained up to 2 h post-injection [37]. The biodistribution profile is characterized by prominent uptake in the kidneys and pancreas, with notably high pancreas-to-blood ratios (14.5 at 60 min), while lower accumulation was observed in the liver, intestines, stomach, and muscle tissues [37]. Renal retention in rodents follows a characteristic temporal pattern, with 16.3% and 5.0% of the injected dose retained at 1 and 3 h, respectively [42].

The high renal uptake observed across species [37,41] represents a limitation for tumor detection in retroperitoneal and pelvic regions. Mechanistic investigations by Wei et al. identified organic anion transporters (OAT1, OAT10, and OCTN2) as key mediators of renal tubular uptake in vitro [43]. The role of OAT1 was confirmed through pharmacological inhibition studies using probenecid, which significantly reduced renal accumulation while increasing plasma levels of FAMT in mice [44]. Subsequent in vivo studies in mice demonstrated that 2-fluoro-α-methyltyrosine, a LAT1-specific tracer with minimal OAT1 affinity, exhibited significantly reduced renal accumulation and enhanced tumor uptake compared to FAMT [44]. These findings highlight the impact of transporter selectivity on biodistribution and support the development of analogs with improved diagnostic profiles.

These pharmacokinetic properties, particularly the rapid blood clearance, high urinary excretion, and low metabolism, contribute to [^18^F]FAMT’s favorable tumor-to-background contrast in PET imaging. 

### 3.5. Clinical Value of [^18^F]FAMT PET in Differential Diagnosis

**Figure 6:**
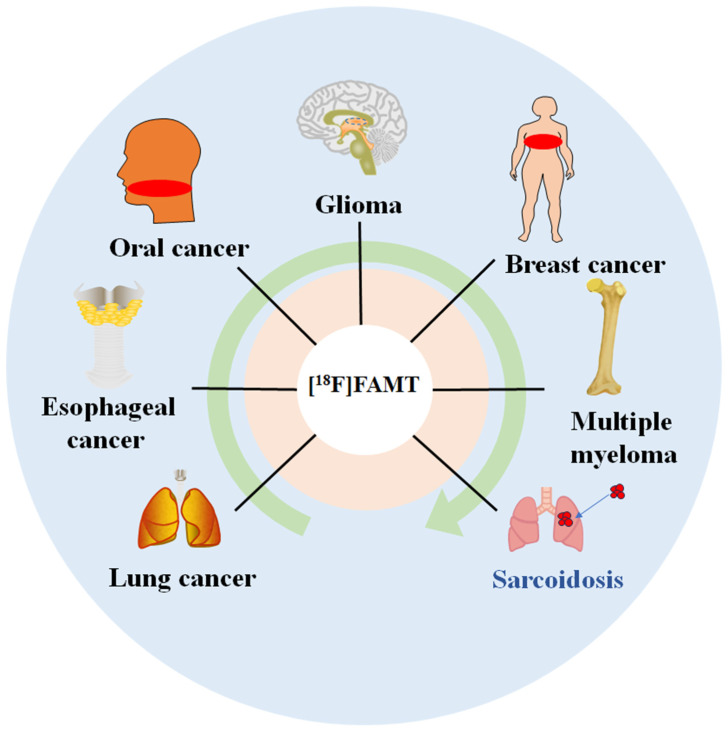
Clinical applications of [^18^F]FAMT PET. [^18^F]FAMT demonstrates diagnostic value in various malignancies (oral cancer, glioma, breast cancer, multiple myeloma, lung cancer, and esophageal cancer) and shows utility in differentiating malignant lesions from inflammatory conditions (sarcoidosis).

Accurate tumor diagnosis is crucial for understanding malignant tumor development, progression, and metastasis. [^18^F]FAMT PET, while showing slightly lower overall sensitivity in malignant lesion detection compared to [^18^F]FDG PET, demonstrates superior specificity and positive predictive value, as shown in Table 1, particularly in distinguishing malignant lesions from inflammatory conditions. [^18^F]FAMT PET has demonstrated diagnostic value in a wide range of tumor types, as shown in Figure 6, offering a complementary approach to existing radiopharmaceuticals in clinical oncology.

Methodology

1. This prospective clinicopathological study enrolled 50 patients with non-small cell lung cancer (NSCLC). Dual-tracer PET imaging was performed using [^18^F]FMT (4–5 MBq/kg) and [^18^F]FDG (5–6 MBq/kg), acquired on a SET 2400 W whole-body PET scanner (Shimadzu, Kyoto, Japan) with BGO crystals. Images were reconstructed using the ordered-subsets expectation maximization (OSEM) algorithm. Standardized uptake values (SUVs) were calculated as follows: SUV = (radioactive concentration in the ROI [MBq/g])/(injected dose [MBq]/body weight [g]). Immunohistochemical analysis evaluated LAT1 expression (affinity-purified rabbit anti-human LAT1 polyclonal antibody; positivity defined as >25% membranous staining) and Ki-67 labeling index (MIB-1 monoclonal antibody). Statistical analysis employed Spearman’s rank correlation and Fisher’s exact test (StatView J-4.5; significance threshold). For full protocol details, see Kaira K, et al.’s Fluorine-18-α-methyltyrosine PET for diagnosis and staging of lung cancer: A clinicopathologic study [45].

2. This clinicopathological study enrolled 37 patients with non-small cell lung cancer (NSCLC) who underwent preoperative PET scans using both 18F-FMT and 18F-FDG tracers. PET imaging was performed with a Shimadzu SET 2400 W scanner, and SUVs were calculated for tumor metabolic activity assessment. Immunohistochemical analyses of resected tumor specimens evaluated angiogenesis markers (VEGF, CD31, CD34), amino acid transporter LAT1, and proliferative activity (Ki-67 labeling index). VEGF expression was quantified as the percentage of positively stained tumor cells, while microvessel density (MVD) was determined via CD31/CD34 hotspot counting. Statistical correlations between PET uptake, clinicopathologic variables, and molecular markers were analyzed using Spearman’s rank test and Fisher’s exact test. The study protocol was approved by the Institutional Review Board of Gunma University, and written informed consent was obtained from all participants.

3. This study employed a prospective design utilizing dual-tracer PET imaging with both ^18^F-FMT (L-[3-^18^F]-α-methyltyrosine) and ^18^F-FDG (^18^F-fluorodeoxyglucose) in 98 patients with stages I–IV NSCLC. Tracer uptake in primary tumors was quantified using the maximal standardized uptake value (SUV_max_), with optimal prognostic cutoffs determined via receiver operating characteristic (ROC) analysis (1.6 for ^18^F-FMT and 11.0 for ^18^F-FDG). Survival outcomes (overall survival [OS] and disease-free survival [DFS]) were analyzed using the Kaplan–Meier method and log-rank test. Multivariate Cox proportional hazards models assessed independent prognostic factors, adjusting for clinical variables (e.g., stage, histology). PET imaging was performed on a Shimadzu SET 2400W scanner, and statistical analyses were conducted using JMP 8 (SAS Institute Inc.) software. For detailed protocols, see Kaira et al., [34].

4. This retrospective study enrolled 21 consecutive patients with histologically confirmed operable esophageal squamous cell carcinoma (SCC) who underwent preoperative staging with CT, ^18^F-FDG-PET, and ^18^F-FAMT-PET. PET/CT scans were performed using Discovery STE (GE Healthcare) or Biograph 16 (Siemens) scanners 60 min after intravenous injection of 5–6 MBq/kg of ^18^F-FAMT or ^18^F-FDG. Images were reconstructed using ordered-subset expectation-maximization algorithms, and standardized uptake values (SUV_max_) were calculated. Lymph node groups were classified according to Japanese Society for Esophageal Disease (JSED) guidelines and evaluated by two blinded nuclear medicine physicians. CT-defined metastases required short-axis lymph node size > 1 cm. The sensitivity, specificity, accuracy, positive predictive value (PPV), and negative predictive value (NPV) were calculated using histopathological results as the gold standard. Statistical analysis included ANOVA for SUV-clinicopathological correlations. Institutional review board approval and written informed consent were obtained.

5. This prospective study enrolled 25 patients with histologically confirmed oral squamous cell carcinoma (OSCC). Both ^18^F-FAMT PET and ^18^F-FDG PET scans were performed preoperatively within a 4-week interval using a Discovery STE PET/CT scanner (GE Healthcare). Tracer uptake was semi-quantitatively analyzed using maximal standardized uptake values (SUV_max_). Immunohistochemical staining with MIB-1 antibody was performed on resected tumor specimens to determine the Ki-67 labeling index (Ki-67 LI), with proliferative activity quantified as the percentage of positively stained nuclei among 1000 counted cells. Spearman’s rank correlation coefficient was used to evaluate associations between SUV_max_ values and Ki-67 LI. Statistical analyses were conducted using Microsoft Excel 2007 and SPSS. Ethical approval and informed consent were obtained, and all protocols followed institutional guidelines.

6. This retrospective study analyzed 68 patients with histologically confirmed OSCC who underwent both ^18^F-FAMT and^18^F-FDG PET imaging. PET scans were performed using a Discovery STE scanner (GE Healthcare) 50 min post-injection of 5 MBq/kg tracer. Semi-quantitative analysis included maximal standardized uptake value (SUV_max_) measurements. Immunohistochemistry assessed LAT1, CD98, Ki-67, CD34, and p53 expression in resected tumor specimens (n = 39), with staining intensity scored on a 4-tier scale. Microvessel density was quantified via CD34-positive vessel counts. Statistical analyses employed Student’s *t*-test, Spearman’s rank correlation, and GraphPad Prism 4/JMP 8 (SAS, Institute, Cary, NC, USA) software. Ethical approval and informed consent were obtained (Gunma University Hospital IRB). For full protocol details, refer to the original article [32].

7. This retrospective study included 27 patients with OSCC who underwent preoperative staging with MRI, ^18^F-FDG PET/CT, and ^18^F-FAMT PET/CT. Imaging findings were compared against histopathological results from surgical specimens as the reference standard. Bone marrow invasion was graded using a 5-point scoring system (0–4) for each modality. Diagnostic performance metrics (sensitivity, specificity, accuracy, PPV, NPV) were calculated. Tumor volumes derived from ^18^F-FAMT and ^18^F-FDG PET/CT were analyzed using PET-VCAR software (GE Healthcare, Milwaukee, WI, USA) with SUV thresholds of 1.4 and 3.0, respectively, and compared to pathological volumes. Statistical analysis employed McNemar’s test for diagnostic accuracy and paired Student’s *t*-test for volume comparisons. MRI interpretations were performed by a single radiologist blinded to PET/CT results, while PET/CT images were reviewed by consensus among nuclear medicine physicians and surgeons.

8. This retrospective study was conducted with approval from the Institutional Review Board of Gunma University Graduate School of Medicine. Informed consent was obtained from all 38 consecutive patients with histologically confirmed gliomas (WHO grades II–IV). Both ^18^F-FAMT and ^18^F-FDG PET scans were performed preoperatively using a Discovery STE (GE Healthcare) or Biograph 16 (Siemens, Munich, Germany) scanner, with three-dimensional acquisition and reconstruction via ordered-subset expectation maximization. Tumor metabolic activity was quantified using the maximum standardized uptake value (SUV_max_) and tumor-to-normal brain ratio (T/N ratio), analyzed independently by nuclear physicians. Proliferation indices (MIB-1 labeling) were assessed immunohistochemically. Statistical analyses included non-parametric tests (Mann–Whitney U, Wilcoxon signed-rank), Spearman’s correlation, and ROC curve analysis (SPSS v21).

9. This retrospective study evaluated 21 patients with bone metastases from various cancers who underwent both ^18^F-FDG and ^18^F-FAMT PET/CT scans within a 1-month interval. Imaging was performed using a Discovery STE (GE Healthcare) or Biograph 16 (Siemens) PET/CT scanner, with 5 MBq/kg tracer doses administered intravenously. Scans were acquired 60 ± 5 min post-injection, reconstructed using 3D ordered subsets expectation maximization, and analyzed for SUV_max_ via manually drawn ROIs. Lesions were selected based on ^18^F-FDG uptake (SUV_max_ ≥ 1.9) and confirmed as metastases through clinical/imaging follow-up. Statistical analysis included linear regression, Mann-Whitney U tests, and *t* tests to compare tracer uptake across lesion types. Institutional ethics approval was obtained for retrospective data analysis.

10. This prospective study enrolled 11 patients with active multiple myeloma (3 untreated, 8 relapsed) who underwent whole-body ^18^F-FAMT and ^18^F-FDG PET scans within a 2-week interval. Spinal MRI was performed to assess bone marrow infiltration patterns. Semi-quantitative analysis included the maximal standardized uptake value (SUV_max_), lesion-to-bone marrow (L/B) ratio, and lesion-to-mediastinum (L/M) ratio. Tracer uptake and ratios were compared using the Student’s *t*-test, with linear regression evaluating correlations between SUV_max_ values. Two blinded nuclear medicine physicians interpreted PET images, with ROIs placed on tracer-avid lesions and background regions (normal bone marrow and aortic arch) for analysis.

#### 3.5.1. Lung Cancer

Non-small cell lung cancer (NSCLC), arising from bronchial mucosa or glands, represents the predominant form of lung malignancy. [^18^F]FAMT PET exhibits remarkable diagnostic performance in primary tumor detection, achieving sensitivity rates of up to 90% [45]. Histopathologic subtype analysis reveals that [^18^F]FAMT shows characteristically higher uptake and sensitivity in squamous cell carcinoma and large cell carcinoma compared to adenocarcinoma [34,45].

##### Diagnostic Performance

In lymphatic staging, [^18^F]FAMT PET demonstrates a unique combination of moderate sensitivity and high specificity [35]. Notably, differential uptake patterns have been observed across NSCLC subtypes, with squamous cell carcinoma (SCC) showing consistently higher tracer accumulation compared to adenocarcinoma (AC) [45]. Compared to [^18^F]FDG PET, [^18^F]FAMT shows distinct advantages in discriminating between malignant lesions and inflammatory processes, particularly in regions with post-treatment changes or granulomatous diseases [6]. In inflammatory conditions, [^18^F]FAMT demonstrates significantly lower uptake compared to [^18^F]FDG, enabling more accurate differentiation of tumor recurrence from post-treatment inflammation [52].

##### Prognostic Significance in NSCLC

The prognostic utility of [^18^F]FAMT extends across various NSCLC subtypes. The maximum standardized uptake value (SUV_max_) serves as a critical prognostic indicator [53,54,55], with a cutoff value of 1.6 significantly correlating with patient outcomes [34]. Higher SUV_max_ values consistently associate with reduced disease-free survival (DFS) rates [34]. Multivariate analyses have validated elevated SUV_max_ as an independent predictor of poor prognosis, particularly in adenocarcinoma patients [34,53]. Given its success in lung cancer imaging and the advantage of minimal cardiac background uptake, [^18^F]FAMT PET has been extensively evaluated in esophageal cancer.

##### Methodological Challenges and Optimization Strategies in NSCLC

In NSCLC imaging, both [^18^F]FAMT and [^18^F]FDG PET/CT are used for assessing tumor metabolism. However, [^18^F]FAMT offers significantly higher specificity, owing to its LAT1-mediated uptake, which correlates closely with proliferation activity (e.g., Ki-67 index) and metabolic tumor volume (MTV). In contrast, [^18^F]FDG, due to its reliance on glucose metabolism, is frequently confounded by inflammatory uptake, especially in post-treatment settings. Quantitative parameters such as SUV_max_ and MTV derived from [^18^F]FAMT imaging have shown superior consistency in distinguishing stable versus progressive disease. MTV in particular has emerged as a potential independent prognostic marker. Nevertheless, technical challenges exist: variability in PET acquisition protocols, scanner models, reconstruction algorithms, and thresholding methods hamper comparability across studies. Manual tumor delineation introduces subjectivity, while partial volume effects reduce measurement accuracy in small lesions.

Histological heterogeneity also poses a challenge. Squamous cell carcinomas typically exhibit high LAT1 expression and consequently higher [^18^F]FAMT uptake, whereas adenocarcinomas show lower uptake despite a stronger correlation with Ki-67. In small cell lung cancer (SCLC), [^18^F]FAMT uptake has been observed to correlate with chemotherapeutic response in limited early investigations, although findings are constrained by small sample sizes. Compared to [^18^F]FDG, which shows strong uptake in inflammatory foci and may overestimate treatment efficacy, [^18^F]FAMT provides a more tumor-specific signal. This makes it particularly advantageous for monitoring therapeutic response in advanced or metastatic disease, where inflammation-related confounders are common. However, its prognostic value in early-stage NSCLC remains uncertain due to limited tumor burden and LAT1 expression variability.

To advance methodological robustness, future studies should aim to standardize [^18^F]FAMT interpretation criteria (current SUV_max_ thresholds range from 1.2 to 1.8 with no consensus), calibrate imaging protocols across centers, and incorporate hybrid imaging modalities (e.g., PET/MRI) to enhance spatial resolution and detect small-volume disease. The use of LAT1 immunohistochemistry could further strengthen imaging–histology correlations. Despite current reliance on small, single-center studies, [^18^F]FAMT PET/CT shows promise in reducing false-positive findings due to inflammation and in accurately quantifying tumor metabolic burden. Its clinical utility will benefit from validation in large-scale, multicenter, and cross-platform investigations. 

#### 3.5.2. Esophageal Cancer

Esophageal cancer remains a significant clinical challenge, with poor prognosis despite advances in surgical techniques and adjuvant therapy. [^18^F]FAMT PET has emerged as a valuable tool in its management, offering insights into diagnosis, staging, and treatment response prediction [47].

##### Diagnostic Accuracy and Staging Utility

Primary tumor detection with [^18^F]FAMT PET demonstrates a sensitivity of 76.2% in esophageal cancer [47]. Pre-treatment studies have shown that 95% of patients exhibit [^18^F]FAMT uptake in primary tumors. Notably, significant correlations exist between [^18^F]FAMT uptake and clinicopathological factors, including depth of invasion, lymphatic invasion, and disease stage [56].

##### Lymph Node Assessment

In the evaluation of lymph node metastasis, [^18^F]FAMT PET exhibits superior specificity compared to both [^18^F]FDG PET and CT, particularly in operable esophageal squamous cell carcinoma [57]. The diagnostic performance achieves 18.2% sensitivity, 100% specificity, and 71.9% overall accuracy [47]. The prognostic significance of lymph node involvement is evident in survival outcomes [57], with patients having one or no metastatic lymph nodes showing a 33.38% five-year survival rate, while those with two or more metastatic nodes demonstrate a markedly reduced rate of 9.35%.

##### Predictive Value for Treatment Outcomes

[^18^F]FAMT uptake patterns demonstrate predictive value for treatment outcomes. Patients exhibiting low [^18^F]FAMT accumulation show significantly higher complete response (CR) rates to chemoradiotherapy compared to those with high accumulation, establishing [^18^F]FAMT uptake as a potential predictor of treatment response [53].

Beyond thoracic malignancies, [^18^F]FAMT PET has shown significant value in head and neck cancers, where imaging faces unique challenges due to complex anatomy and high metabolic background [58,59].

##### Critical Analysis of Methodological Challenges in ESCC

In ESCC, [^18^F]FAMT PET/CT offers distinct methodological advantages over [^18^F]FDG, primarily due to its LAT1-specific uptake and reduced sensitivity to inflammatory changes. This results in significantly higher specificity in lymph node assessment—reaching up to 100%—compared to [^18^F]FDG, which frequently yields false-positives in inflammatory or post-treatment settings. While [^18^F]FAMT SUVmax correlates robustly with pathological features such as depth of invasion, nodal metastasis, and advanced stage, [^18^F]FDG-derived SUV_peak_ has shown greater relevance in predicting short-term disease-free survival, albeit with lower specificity.

The characteristic pattern of multi-regional lymphatic spread and predominant squamous histology in ESCC further supports the utility of [^18^F]FAMT, especially when contrasted with tumor types exhibiting low LAT1 expression (e.g., lung adenocarcinoma). However, the limited sensitivity of [^18^F]FAMT for small-volume lesions underscores the need for multimodal imaging approaches, particularly in early-stage disease or preoperative staging. 

Standardization of imaging protocols remains a key methodological challenge. Variability in SUV thresholds (e.g., 1.2–1.8) and acquisition settings across studies limits comparability and clinical application. While amino acid tracers like [^18^F]FAMT offer superior prognostic value and reduced inflammation-related uptake, prospective validation and harmonized quantification criteria are urgently needed. A combined imaging strategy integrating [^18^F]FAMT with [^18^F]FDG may help balance sensitivity and specificity, thereby enabling more accurate risk stratification and treatment planning in ESCC.

#### 3.5.3. Oral Cancer

Oral cancer accounts for approximately 2% of global cancer diagnoses, with particularly high prevalence in developing nations. Over 90% of oral cancers manifest as squamous cell carcinomas originating from mucous membrane linings [48]. The complex anatomy of the head and neck region poses significant challenges for conventional diagnostic methods in accurately assessing tumor infiltration [60].

##### Diagnostic Efficacy in Oral Cancer

[^18^F]FAMT PET demonstrates exceptional diagnostic capabilities in oral cancer, with primary tumor detection sensitivity reaching 98%. In lymph node evaluation, it achieves 68% sensitivity, 99% specificity, and 97% accuracy [32]. [^18^F]FAMT PET/CT demonstrates superior diagnostic accuracy compared to [^18^F]FDG PET/CT in detecting malignant lymph nodes (accuracy: 97% vs. 84%), particularly valuable in surgical planning for minimizing resection extent [32]. This higher accuracy is particularly valuable in post-treatment surveillance, where [^18^F]FAMT shows minimal uptake in inflammatory changes, unlike [^18^F]FDG [52].

##### Correlation with LAT1 and Prognosis

In oral cancer tissue, [^18^F]FAMT uptake shows significant correlations with LAT1 expression and tumor proliferation [32]. This correlation provides valuable information for surgical planning, enabling more precise determination of tumor margins and potentially reducing the extent of surgical resection while maintaining oncological safety [52].

While [^18^F]FAMT has shown considerable utility in various peripheral tumors, its application in central nervous system malignancies presents unique opportunities and challenges, particularly in gliomas, where conventional imaging modalities often have limitations.

##### Methodological Challenges and Optimization Strategies in OSCC

In OSCC imaging, critical methodological differences between [^18^F]FAMT and [^18^F]FDG PET/CT reflect their distinct physiological mechanisms and clinical performance. [^18^F]FDG, based on glucose metabolism, offers high sensitivity for primary tumors but demonstrates limited specificity in lymph node assessment due to inflammatory uptake—particularly problematic in tongue tumors. Its SUV is influenced by intratumoral inflammation and does not reliably correlate with molecular markers such as Glut1. In contrast, [^18^F]FAMT exhibits high tumor selectivity by targeting L-type amino acid transporter 1 (LAT1), achieving 98% sensitivity for primary lesions and up to 99% specificity in non-tongue nodal metastasis. Its SUV_max_ correlates significantly with LAT1 expression, Ki-67 index, and tumor stage, aligning more closely with tumor aggressiveness and proliferative activity. These attributes improve diagnostic accuracy, especially in post-treatment settings where inflammatory changes can obscure true recurrence on [^18^F]FDG imaging.

Despite these advantages, [^18^F]FAMT presents limitations: lower sensitivity for small lesions, lack of standardized SUV cut-offs, and insufficient multicenter validation currently hinder widespread clinical adoption. Anatomical and histological heterogeneity, such as reduced LAT1 expression in adenocarcinomas, further affect tracer uptake patterns.

Overall, the integration of [^18^F]FAMT with [^18^F]FDG may provide complementary diagnostic value—balancing metabolic sensitivity and molecular specificity—and support more accurate treatment planning in OSCC.

#### 3.5.4. Glioma

Glioma, characterized by diffuse glial cell proliferation, presents unique diagnostic challenges among primary brain tumors. [^18^F]FAMT PET has demonstrated utility in glioma imaging, offering advantages over conventional imaging modalities [61].

##### Diagnostic Performance

[^18^F]FAMT PET effectively distinguishes between high-grade gliomas (HGGs) and low-grade gliomas (LGGs) through tumor-to-normal tissue (T/N) ratios. Median T/N ratios are 2.85, 4.65, and 4.09 for grade II, III, and IV gliomas, respectively. An optimal cutoff value of 3.37 achieves 81% sensitivity, 67% specificity, and 76% accuracy in differentiating HGGs from LGGs [49].

##### Comparative Advantages

[^18^F]FAMT PET demonstrates superior tumor delineation compared to MRI or CT imaging, particularly in low-grade gliomas and gliomatosis cerebri [49]. Notably, [^18^F]FAMT achieves significantly higher T/N ratios compared to [^18^F]FDG across all glioma subtypes, providing enhanced contrast and clearer visualization [61]. In low-grade gliomas, where [^18^F]FDG often shows limited utility due to high physiological brain uptake [49], [^18^F]FAMT demonstrates superior detection capability with tumor-to-background ratios significantly higher than [^18^F]FDG. This advantage is particularly pronounced in cortical regions where [^18^F]FDG’s high physiological uptake often masks tumor presence.

##### Biological and Methodological Challenges in Glioma Imaging

The application of [^18^F]FAMT PET in glioma imaging introduces both notable advantages and inherent methodological challenges. One of the most critical limitations in brain tumor imaging lies in the high physiological uptake of glucose in normal brain tissue, which severely compromises the specificity of [^18^F]FDG PET. Although [^18^F]FDG is sensitive to metabolic activity, it frequently yields false negatives in low-grade gliomas (LGGs) due to low proliferative activity and confounding uptake from inflammation or non-neoplastic processes. In contrast, [^18^F]FAMT offers improved tumor-to-background contrast by specifically targeting the LAT1 transporter, which is upregulated in gliomas but minimally expressed in healthy brain tissue. This molecular selectivity results in significantly higher T/N ratios, particularly in high-grade gliomas (HGGs), and improves the detection of LGGs, which are often poorly visualized with [^18^F]FDG. The higher detection rate of LGGs using [^18^F]FAMT (95% with T/N > 2.0) compared to [^18^F]FDG (39%) illustrates this clear diagnostic advantage. 

However, methodological limitations of [^18^F]FAMT remain. The tracer may exhibit reduced sensitivity in detecting small or infiltrative lesions, especially those with low LAT1 expression. Furthermore, the absence of standardized SUV or T/N cutoff values across studies limits comparability and hinders clinical decision-making. Dynamic PET imaging, which captures tracer kinetics, may enhance tumor grading performance, but its implementation remains limited and technically demanding.

When compared to other amino acid tracers, such as ^11^C-MET and [^18^F]FET, [^18^F]FAMT demonstrates a favorable profile with a longer half-life and higher LAT1 specificity, supporting broader clinical application and more biologically meaningful interpretation. Nonetheless, its relatively lower absolute uptake and limited availability currently restrict routine use in many centers.

Unlike extracranial malignancies, gliomas present additional imaging complexities due to the presence of the blood–brain barrier (BBB), particularly in non-enhancing or LGG lesions. These unique pathophysiological features necessitate the use of tracers like [^18^F]FAMT that can reflect underlying tumor biology with high specificity. Moreover, the potential complementary use of [^18^F]FAMT with [^18^F]FDG may allow for a dual evaluation of tumor metabolism and molecular specificity, particularly in differentiating tumor recurrence from treatment-related changes such as radiation necrosis.

Taken together, [^18^F]FAMT PET represents a biologically rational and technically promising approach for glioma imaging, particularly in distinguishing tumor grade and delineating tumor margins. Nevertheless, current methodological limitations—including the need for dynamic protocols, standardized uptake thresholds, and multicenter validation—must be addressed before its broader clinical integration can be realized.

#### 3.5.5. Applications in Other Tumors and Sarcoidosis

##### Multiple Myeloma

Multiple myeloma (MM), characterized by monoclonal plasma cell proliferation in bone marrow, presents unique diagnostic challenges. [^18^F]FAMT PET has emerged as a valuable tool for detecting active multiple myeloma lesions, with its uptake closely correlating with tumor cell proliferative activity, enabling effective differentiation between benign and malignant lesions [51].

##### Diagnostic Specificity and Imaging Challenges in MM

In multiple myeloma (MM), [^18^F]FDG PET is widely used but often yields false-positive results due to inflammation or bone marrow hyperplasia, complicating lesion interpretation. [^18^F]FAMT PET offers higher specificity by targeting LAT1 expression, which is closely associated with tumor proliferation rather than inflammatory activity. Comparative data show [^18^F]FAMT detects 87.5% of [^18^F]FDG-positive lesions while providing improved lesion characterization. However, standardized uptake thresholds and prospective studies remain lacking. Notably, the combined use of [^18^F]FAMT and [^18^F]FDG may offer synergistic diagnostic value by integrating metabolic activity with molecular specificity, but its clinical implementation requires harmonized imaging protocols.

##### Bone Metastases

Studies have revealed distinctive uptake patterns of [^18^F]FAMT in bone metastases, with higher uptake observed in squamous cell carcinoma compared to adenocarcinoma [38,50]. Notably, [^18^F]FAMT uptake shows no significant differences between osteoblastic and osteolytic metastases, suggesting its potential utility in comprehensive bone metastasis evaluation [50].

##### Diagnostic Specificity and Phenotype-Related Variation in Bone Metastases

Bone metastases represent a heterogeneous group of lesions, including osteolytic and osteoblastic subtypes, with variable tracer behavior. [^18^F]FDG PET typically shows higher uptake in osteolytic lesions but has limited sensitivity for osteoblastic metastases, leading to underdiagnosis. In contrast, [^18^F]FAMT exhibits more consistent uptake regardless of metastatic subtype, achieving high specificity (97.4%) and enabling clearer delineation in squamous cell carcinoma metastases. Importantly, the [^18^F]FAMT/FDG uptake ratio differs by histology (0.52 for squamous cell carcinoma vs. 0.35 for adenocarcinoma), suggesting that tracer performance is influenced by tumor phenotype. Nevertheless, larger cohort studies and kinetic modeling are needed to standardize diagnostic thresholds and assess response monitoring utility. 

##### Breast Cancer

[^18^F]FAMT PET has demonstrated promising results in breast cancer imaging, particularly in the detection and characterization of primary breast tumors. In MCF-7 xenograft models, [^18^F]FAMT has shown high specificity for tumor detection with favorable tumor-to-background ratios. Comparative studies with [^18^F]FDG in breast cancer have shown that while [^18^F]FDG demonstrates higher sensitivity in detecting primary tumors, [^18^F]FAMT exhibits superior specificity in differentiating malignant from benign lesions [42]. This complementary pattern suggests potential value in using both tracers for comprehensive breast cancer evaluation.

##### Critical Appraisal of Specificity and Prognostic Imaging in Breast Cancer

In breast cancer, [^18^F]FDG PET remains highly sensitive for primary and metastatic lesions but may lack specificity in differentiating malignancy from benign inflammatory processes. [^18^F]FAMT PET demonstrates improved specificity—particularly in low-background regions such as the left breast and liver—due to minimal cardiac and hepatic uptake. The tumor-to-muscle (T/M) ratio of [^18^F]FAMT strongly correlates with the Ki-67 index, providing added value in assessing tumor proliferation. These features highlight [^18^F]FAMT’s potential in grading and prognostication. However, its lower absolute sensitivity compared to [^18^F]FDG necessitates a combined diagnostic strategy. Current limitations include small sample sizes, lack of multicenter validation, and absence of standardized acquisition protocols. 

##### Sarcoidosis

[^18^F]FAMT demonstrates unique characteristics in sarcoidosis imaging. While sarcoidosis lesions exhibit high [^18^F]FDG uptake, they show minimal [^18^F]FAMT accumulation [62]. This differential uptake pattern suggests that combining [^18^F]FAMT with [^18^F]FDG PET could enhance the accuracy of distinguishing between sarcoidosis and malignant lesions.

##### Critical Analysis of Diagnostic Imaging in Sarcoidosis

While [^18^F]FDG is highly sensitive for detecting inflammatory lesions such as sarcoid granulomas, it lacks specificity in distinguishing them from malignancies like lymphoma. In contrast, [^18^F]FAMT exhibits minimal uptake in granulomas but selectively accumulates in lymphoma due to LAT1 overexpression. This complementary uptake pattern—particularly the “[^18^F]FDG-positive/[^18^F]FAMT-negative” signature—can help differentiate residual inflammatory lesions from recurrent malignancy in post-therapy settings, thereby enhancing diagnostic confidence. Nevertheless, current supporting evidence is limited to small case series, and standardized interpretation protocols remain to be established. 

### 3.6. Limitations and Challenges

The development and application of [^18^F]FAMT PET face several significant challenges despite its promising clinical value. These limitations span technical, biological, and practical aspects of its implementation.

#### 3.6.1. Technical Challenges

The production of [^18^F]FAMT presents substantial technical hurdles, with current synthesis processes suffering from limited radiochemical yield and complex procedures that resist automation [13]. The inability to produce [^18^F]FAMT using automated synthesizers at most PET facilities, coupled with the relatively short half-life of ^18^F (110 min), significantly restricts its widespread clinical implementation, particularly for centers without on-site cyclotron facilities [63].

#### 3.6.2. Biological and Imaging Limitations

Several biological factors affect [^18^F]FAMT performance as a PET tracer. The variability in LAT1 expression across different tumor types and stages impacts imaging sensitivity, while the moderate sensitivity in certain cancer types, particularly in lymph node staging, may necessitate complementary imaging methods [35]. The optimization of imaging acquisition timing and interpretation protocols remains crucial for maximizing diagnostic accuracy.

#### 3.6.3. Clinical Implementation and Standardization Issues

The clinical adoption of [^18^F]FAMT PET faces several challenges related to standardization and practical implementation. These include the need for consistent production methods, standardized SUV thresholds, and uniform image acquisition protocols across institutions. Compared to widely used PET tracers such as [^18^F]FDG, [^18^F]FAMT offers the advantage of LAT1 specificity, making it a promising candidate for tumor imaging with reduced nonspecific uptake. However, its clinical translation has been constrained by technical challenges in radiosynthesis, including the reliance on electrophilic [^18^F]fluorination and relatively low radiochemical yields, which may limit cost-effectiveness and scalability. Furthermore, [^18^F]FAMT exhibits significant renal retention, which complicates the detection of tumors in abdominal and pelvic regions. In contrast, [^18^F]FDG benefits from well-established production protocols and widespread clinical availability, despite its high background uptake in inflammatory and normal tissues.

## 4. Conclusions

[^18^F]FAMT has emerged as a promising amino acid PET tracer for tumor imaging, distinguished by its unique structural characteristics and high tumor specificity. The selective transport of [^18^F]FAMT via LAT1, coupled with its favorable pharmacokinetic profile, particularly its rapid blood clearance and low physiological background, makes it valuable for tumor diagnosis and assessment. Although [^18^F]FAMT initially showed good potential as an LAT1-specific tracer, its technical limitations (synthetic challenges, renal retention) [64], its problems with cost and toxicity have led to a gradual decline in [^18^F]FAMT research in recent years.

Several key advantages of [^18^F]FAMT have been demonstrated across various clinical applications. In lung cancer and esophageal cancer, it shows particular value in lymph node staging with high specificity. For oral and head and neck cancers, its superior tumor-to-background contrast aids surgical planning. In brain tumors, especially gliomas, [^18^F]FAMT demonstrates advantages over conventional tracers due to its low physiological brain uptake. The correlation between [^18^F]FAMT uptake and tumor proliferation markers provides a molecular basis for assessing tumor aggressiveness and monitoring treatment response.

Future research in [^18^F]FAMT should pursue technical advancement through improved synthesis methods and automated production processes, while optimizing imaging protocols for specific clinical scenarios. Clinical applications need to be expanded through validation studies across different tumor types, with emphasis on establishing standardized quantitative criteria for response assessment and investigating its role in treatment planning. Further exploration of the relationship between LAT1 expression and [^18^F]FAMT uptake, along with the development of novel analogues, will deepen our understanding of its biological mechanisms. Additionally, the unique tumor specificity of [^18^F]FAMT structure presents opportunities for theranostic applications, particularly in the development of LAT1-targeted radiopharmaceuticals for targeted alpha therapy (TAT). Recent studies exploring At-labeled FAMT analogues have demonstrated promising results for TAT applications [65,66], highlighting the potential of FAMT-based compounds in both diagnostic and therapeutic strategies.

The rapid advancements in molecular imaging techniques have positioned [^18^F]FAMT as a promising tool for enhancing the accuracy and specificity of tumor diagnosis. Similarly, 2-(76)Br-BAMP, as an analog of FAMT, represents a promising candidate for developing novel PET tracers in oncologic imaging applications [67]. With ongoing technological refinements and extensive clinical validation, both [^18^F]FAMT and 2-(76)Br-BAMP are poised to assume increasingly prominent roles in the field of tumor imaging. This progression is anticipated to expand the clinical utility of amino acid-based tracers while concurrently advancing the development of personalized therapeutic strategies for cancer treatment.

## Figures and Tables

**Table 1:** Comparative diagnostic performance of [^18^F]FAMT and [^18^F]FDG PET in different cancer types.

Indication	Tumor	Tracer	Refs.	Year	Methodology Ref.	No. of Patients	Sens.	Spec.	SUV
The primary tumor detection	NSCLC	[^18^F]FAMT	[45]	2007	1	50	90%	NR	NR
			[46]	2009	2	37	NR	NR	2.65
			[34]	2009	3	98	NR	NR	0.6–5.8, median of 1.6
		[^18^F]FDG	[45]	2007	1	50	94%	NR	NR
			[46]	2009	2	37	NR	NR	11.5
			[34]	2009	3	98	NR	NR	0.9–29.6, median of 6.8
	ESCC	[^18^F]FAMT	[47]	2010	4	21	76.2%	NR	NR
		[^18^F]FDG	[47]	2010	4	21	90.5%	NR	NR
	OSCC	[^18^F]FAMT	[38]	2010	5	25	84%	NR	1.3–8.5, median of 3.5
			[32]	2013	6	68	98%	NR	NR
			[48]	2013	7	27	90%	85.7%	NR
		[^18^F]FDG	[38]	2010	5	25	88%	NR	4.2–15.9, median of 9.7
			[32]	2013	6	68	100%	NR	NR
			[48]	2013	7	27	100%	14.3%	NR
LN Staging	NSCLC	[^18^F]FAMT	[45]	2007	1	50	57.8%	100%	NR
		[^18^F]FDG	[45]	2007	1	50	65.7%	91%	NR
	ESCC	[^18^F]FAMT	[47]	2010	4	21	18.2%	100%	NR
		[^18^F]FDG	[47]	2010	4	21	24.2%	93.7%	NR
	OSCC	[^18^F]FAMT	[32]	2013	6	68	68%	99%	NR
		[^18^F]FDG	[32]	2013	6	68	84%	94%	NR
	Glioma	[^18^F]FAMT	[49]	2017	8	38	61.5%	75%	0.94
		[^18^F]FDG	[49]	2017	8	38	89%	67%	5.91
	BM	[^18^F]FAMT	[50]	2013	9	21	NR	97.4%	2.88–4.20, median of 3.6
		[^18^F]FDG	[50]	2013	9	21	NR	NR	5.40–9.15, median of 6.55
	MM	[^18^F]FAMT	[51]	2012	10	11	NR	NR	0.80–4.90, median of 1.5
		[^18^F]FDG	[51]	2012	10	11	NR	NR	1.50–7.20, median of 2.8

Abbreviations: Sens., Sensitivity; Spec., Specificity; LN Staging, lymph nodes staging; NR, not reported; SUV, standard uptake value; NSCLC: non-small cell lung cancer; ESCC: esophageal squamous cell carcinoma; OSCC: oral squamous cell carcinoma; BM: bone metastases; MM: multiple myeloma.

## Data Availability

Not applicable.

## Abbreviations

The following abbreviations are used in this manuscript:

[^18^F]FAMT	L-3-[^18^F]-fluoro-α-methyltyrosine
[^18^F]FDG	2-[^18^F]Fluoro-2-deoxy-D-glucose
PET	positron emission tomography
^11^C-MET	I-methyl-^11^C-methionine
[^18^F]FET	O-(2-[^18^F]-fluoroethyl)-L-tyrosine
[^123^I]IMT	3-[^123^I]-L-α-methyl-tyrosine
LAT1	L-type amino acid transporter 1
CH_3_COO^18^F	^18^F-acetylhypofluorite
NSCLC	non-small cell lung cancer
SCC	squamous cell carcinoma
AC	adenocarcinoma
LCC	large cell carcinoma
SUV_max_	maximum standardized uptake value
LN	lymph nodes
CT	computerized tomography
ROC	receiver operating characteristic
HGG	high-grade gliomas
LGG	low-grade gliomas
MM	multiple myeloma
OAT	organic anion transporter
